# Essential key indicators for disaster medical response suggested to be included in a national uniform protocol for documentation of major incidents: a Delphi study

**DOI:** 10.1186/1757-7241-21-68

**Published:** 2013-09-11

**Authors:** Monica Rådestad, Maria Jirwe, Maaret Castrén, Leif Svensson, Dan Gryth, Anders Rüter

**Affiliations:** 1 Department of Clinical Science and Education and Section of Emergency Medicine, Södersjukhuset, Karolinska Institutet, Stockholm, Sweden; 2 Department of Neurobiology, Care Sciences and Society and Division of Nursing, Karolinska Institutet, Stockholm, Sweden; 3 Department of Clinical Science and Education, Karolinska Institutet, Stockholm, Sweden; 4 Department of Physiology and Pharmacology, Section of Anaesthesiology and Intensive care, Karolinska Institutet, Stockholm, Sweden; 5 Sophiahemmet University, Stockholm, Sweden

**Keywords:** Delphi technique, Consensus, Disaster data reporting, Disaster response, Major incident medical management, Utstein-style

## Abstract

**Background:**

Registration of data from a major incident or disaster serves several purposes such as to record data for evaluation of response as well as for research. Data needed can often be retrieved after an incident while other must be recorded during the incident. There is a need for a consensus on what is essential to record from a disaster response. The aim of this study was to identify key indicators essential for initial disaster medical response registration. By this is meant nationally accepted processes involved, from the time of the emergency call to the emergency medical communication centre until medical care is provided at the emergency department.

**Methods:**

A three round Delphi study was conducted. Thirty experts with a broad knowledge in disaster and emergency response and medical management were invited. In this study we estimated 30 experts to be approximately one third of the number in Sweden eligible for recruitment. Process, structure and outcome indicators for the initial disaster medical response were identified. These were based on previous research and expressed as statements and were grouped into eight categories, and presented to the panel of experts. The experts were instructed to score each statement, using a five point Likert scale, and were also invited to include additional statements. Statements reaching a predefined consensus level of 80% were considered as essential to register.

**Results:**

In total 97 statements were generated, 77 statements reached consensus. The 77 statements covered parts of all relevant aspects involved in the initial disaster medical response. The 20 indicators that did not reach consensus mostly concerned patient related times in hospital, types of support systems and security for health care staff.

**Conclusions:**

The Delphi technique can be used for reaching consensus of data, comprising process, structure and outcome indicators, identified as essential for recording from major incidents and disasters.

## Background

There is a need for uniform data reporting of results from medical response management to major incidents (MIs) and disasters in order to make data available for evaluation, quality control, scientific analysis and development [1-4]. Emergency medical service (EMS) documentation is often inadequate, hampering evaluation of disaster medical responses (DMR) and losing data for research [5]. The development of a standard template for registration of essential data from MIs and disasters can be achieved using various methods. Some published reports have used an international panel of researchers as informants, while others have been largely based on personal expert knowledge and experience [6-8]. If we are to improve the impact of the DMR on patient outcome, however, there is a need to develop evidence-based improvements in medical response management [8]. So far, to our knowledge, no published studies on DMR have reported results in recommended format, which emphasizes the gap between research and practice. In spite of international initiatives the need to perform national or regional modifications, in order to develop a template that will be generally accepted and used, remains. The ideal would be for these modifications to be suitable in the national context as well as providing the possibility to contribute to international research. One reason for the delay in accepting an international standard is that there still is a lack of consensus on what DMR data is essential to serve the purposes mentioned above.

The Delphi study technique has been used in several contexts and has also demonstrated the possibility to reach consensus in the field of disaster medicine [8-10]. A Delphi study can be described as mapping of expert opinion in the field of investigation, and it is especially suited for complex issues where difficulties in achieving group consensus exist [11,12].

It is likely that a template based on national consensus is more easily implemented in the form of a quality register. The healthcare authorities of several countries have given priority to the development of national registers to be used as a basis for audit, quality improvement and research. Process indicators, often described as a tool for measuring steps of activities in medical care and often linked to patient outcome [13], have also been used in recent years for deriving criteria used for the assessment of the quality of DMR [5]. The most important outcome in disaster management is the reduction in morbidity and mortality. However, this is a fairly blunt instrument for evaluating the management of individual incidents, and therefore the assessment of processes and structure involved, is important in disaster medicine research. All DMR activities that influence patient outcome must therefore be identified. This approach has also been described in a recently published international consensus study [8].

The aim of this study was to identify key indicators essential for initial DMR registration. By this is meant nationally accepted processes involved, from the time of the emergency call to the emergency medical communication centre (EMCC) until medical care is provided at the emergency department (ED).

### Definitions

**Disaster medical response** (DMR): the collective action taken by all relevant agencies (including the EMS system) immediately, during and after a disaster or MI.

**Duty officer** (DO): In Sweden the DO receives alarm information from the EMCC. In the case of an MI or disaster he/she has the authority to declare “major incident” and initiate the relevant processes at the regional level. The DO also has the responsibility to initially coordinate and thereby act as the initial regional commander for all medical strategies [14].

The DO alerts health-care facilities within the region and sends a *“distribution key”* to the ambulance evacuation officer at the scene of the incident, based on resource capacity reports such as available operating theatres, ICU beds etc.

**Expert**: according to what is stated in the literature, an expert has special knowledge or skill in some particular field, “someone who has knowledge about a specific subject” [12]. In the present study disaster medicine is the particular field.

**Indicators:** in the present study, there are three different kinds of indicators important for measuring DMR management.

**Outcome indicator**: an indicator describing outcome of health care [13], in disaster medical management is the reduction in morbidity and mortality of the disaster survivors the most important outcome.

**Process indicator**: an indicator describing activities or processes [13] involved in medical response management at an MI, and is usually associated with patient outcome.

**Structure indicator:** a quantitative measure reflecting availability of resources [13], for example number of ambulances, involved in medical response management at an MI.

**Major incident**: defined by national regulations as an incident having the magnitude or severity, that resources available are strained and must be managed, organised and used in a special way [14]. This highlights the imbalance between the immediate requirements of medical response management and the immediate access to resources, regardless of type of incident or number of casualties [15].

**Medical management**: a process that involves coordination of health-care facilities and activities according to the national model [14]. MI medical response management encompasses all processes of decision-making at the scene, at the strategic level and at the health-care facility level, and is conducted according to guidelines to assure that the appropriate medical care is provided to all ill/injured patients.

## Methods

A modified Delphi technique, focusing on the initial DMR, was adopted for the present study. The Delphi technique is based on a systematic, iterative collection of expert opinions (not involving face-to-face meetings) to discuss a subject under investigation. The technique employs a series of rounds and begins with a questionnaire or an interview seeking the chosen experts’ opinion and comments on the subject under investigation [16]. After a pilot study involving three teachers in disaster medicine, minor modifications were made to the statements in the questionnaire. Level of agreement of statements or importance of issues between experts is scored and the data can be analysed statistically. Feedback on the results after each round is then given to the members of the expert panel. The Delphi process continues until the investigator no longer anticipates any further increase in statement consensus. During this process there is a risk that some experts lose interest and drop out. There must therefore be a balance between attempting to reach consensus and the risk of losing experts. As a consequence, the number of rounds is often predefined and the literature gives no strict guidelines on this matter [11,16]. In the present study the distribution of questionnaires and collection of data were performed by e-mail and the number of rounds was set at three. Three reminder letters were sent to non-responders after each round.

### Expert group

The Delphi study was conducted between April and November 2012. A strategic selection of experts was made. The study required that the expert should be knowledgeable in disaster response, disaster research and/or medical management of disasters (Table 1). Experts were recruited from both research and practical fields, including researchers, duty officers and representatives from national and regional authorities. For the purpose of analysis these subgroups were treated as a homogenous group (Table 2). In the literature a broad choice of experts from diverse expertise and geographic areas is highly recommended [11] and for this reason we chose a panel consisting of experts from various parts of Sweden. Thirty experts were included. The literature gives no clear recommendation regarding an optimal number of participants [11] and this can vary from a few members up to thousands depending on whether it is a homogenous or heterogeneous group [16]. In this study we estimated 30 experts, approximately one third of the number in Sweden, to be eligible for recruitment. Thirty experts were invited by strategic selection and all accepted to participate. All experts were informed about the study and the estimated time of commitment. This was done via personal contact (face-to-face or telephone) as suggested by McKenna and Jirwe et al. [17,18]. The experts were aware of the others participating in the group, but their response to each questionnaire remained strictly unknown to each other. According to McKenna the Delphi technique cannot guarantee complete anonymity and therefore uses the term “quasi-anonymity” [17]. In the literature this quasi-anonymity is highlighted as a motivation factor for participation and may also increase the response rate [11,12].

**Table 1:** Demographic data of the expert group

		**No**
**Gender**	**Male/female**	**18/12**
**All experts have a position with responsibilities in the field of disaster and/or emergency medicine.**		
	Researcher (MD or RN)	11
	MD other	2
	RN other	6
	Administrator	3
	Manager	8
**Length (years) of experience within expert field**		
	1-5	3
	6-10	-
	11-20	8
	>20	14
	Missing data	5

**Table 2:** Affiliation of the expert group

**Affiliation of the expert group**	**Round 1**	**Round 2**	**Round 3**
**Researchers**	10 (33.3 %)	10 (33.3%)	10 (33.3 %)
**Duty officers (Regional level)**	10 (33.3%)	10 (33.3%)	10 (33.3%)
**Other national/regional authorities**	10 (33.3%)	9 (30%)	9 (30%)
**Total participants**	30 (100 %)	29 (96.6 %)	29 (96.6 %)

### Ethical considerations

Participants were assured of the confidentiality of the information they provided and that their anonymity would be ensured in any reports emanating from the study. The principles stated in World Medical Association Declaration of Helsinki was adhered to as well as Swedish rules regarding ethics approval [19].

### Round 1

The content of the first questionnaire was based on a review of the literature regarding sets of criteria that have been used when developing templates for the collection and reporting of DMR data [6,8]. The first questionnaire, distributed in April 2012, also included a glossary of terms related to DMR. In addition to demographic data (incident characteristics), essential indicators for the initial DMR were listed and expressed as 85 statements. The statements were grouped into eight predefined areas;(1) initial medical response management at the regional (strategic) level; (2) type of incident (incident characteristics); (3) initial medical response management at the local level (at the scene of the incident); (4) management/liaison (in general); (5) patient transport/resources; (6) initial medical response management at the local level (healthcare facilities); (7) injury severity and mortality (patients characteristics); (8) staff equipment (Table 3).

**Table 3:** Statements that reached expert consensus

**Area**	**Mean**	**SD**	**Round**
**Initial medical response management at the regional (strategic) level**			No
Time point of notification of the first appropriate staff person, Duty Officer (DO) to take on the role of medical management coordinator	4.93	0.25	1
Location of incident	4.90	0.30	1
Time point when major incident was declared by duty officer (DO)	4.75	0.64	1
Time point when regional medical command centre establishes contact with management at the scene of the incident	4.75	0.51	1
Content of regional medical command centre issues guidelines for course of action	4.66	0.80	1
Time point for decision on additional recourses to be sent to the scene	4.65	0.61	1
Time point when regional medical command centre issues guidelines for course of action	4.63	0.66	1
Time point for and changes in operational level of regional medical command centre	4.62	0.67	3
Who reported incident	4.62	0.62	1
Time point and if other person than DO assumes the role as initial medical management coordinator	4.55	0.82	3
Operational level of regional medical command centre	4.44	1.05	1
Time point at which the regional medical command centre deactivates its DMMP	4.42	1.10	2
**Type of incident (incident characteristics)**			No
Incident type	4.88	0.42	2
**Initial medical response management at the local level (scene of the incident)**			No
Time for first report from scene “Window report”	4.89	0.40	1
Content of the first management at the incident site, decisions about the course of action/issues guidelines for the medical response	4.85	0.35	2
First EMS ambulance at the incident site and time of arrival	4.82	0.66	1
Number of mobile medical teams and time point when alarming each one	4.79	0.67	1
Time points at which medical responders at scene are activated and demobilised	4.78	0.49	3
Content of second report from scene	4.77	0.69	2
The number of T1 category patients transported from the scene to receiving healthcare facilities	4.77	0.80	1
Content, according to METHANE, of first report from scene according to the MIMMS concept *	4.75	0.58	2
Content of the first management at the incident site, decisions about the course of action/issues guidelines for response	4.74	0.52	3
Number of ambulances and time point for alarming each ambulance	4.73	0.90	1
Number of first responder vehicles and time point for alarming each	4.46	1.10	1
Number of ambulance helicopters alarmed and time point for alarming each	4.76	0.67	1
Time point when on scene medical management establishes contact with regional medical command centre	4.68	0.60	1
Time point when on scene medical control and coordination is operational	4.67	0.72	1
The number of T2 category patients transported from the scene to receiving healthcare facilities	4.65	0.84	1
Time point when first EMS ambulance arrives on scene	4.57	0.79	2
Time point when on scene medical control and coordination is demobilised	4.57	0.74	3
The number of T3 category patients transported from the scene to receiving healthcare facilities	4.55	0.93	1
Time for second report from scene “confirming report”	4.53	0.96	1
Time point when management at the incident site makes decisions about the course of action/ issues, guidelines for the medical response	4.53	0.99	3
Time when management at the incident site issues the first course of action, general guidelines for response	4.51	1.01	1
The number of T4 category patients transported from the scene to receiving healthcare facilities	4.44	0.78	3
Units sending prehospital teams	4.41	0.73	3
Time point at which the first patient has been triaged at the scene of the MI	4.37	0.86	3
Triage system used	4.35	0.91	3
Time when casualty-clearing station can start receiving patients	4.34	0.72	3
**Management/liaison (in general when nothing else is specified)**			No
Time point for the first referral of patients to the receiving health care facilities (referral key)	4.65	0.72	1
Time point when contact made with the authority at the national level	4.65	0.61	3
Time for decision if another county / region needs to be alerted and content of this decision	4.63	0.80	1
Time point for the first decision on the course of action at the regional level of management	4.62	0.62	1
The measures initiated to address operational /infrastructure disruption	4.60	0.78	3
What kind of expertise becomes engaged	4.55	0.63	3
Decision on information to “own staff” in the county health care units	4.53	0.69	3
Time point when liaison is established with other actors, emergency support function (ESP)	4.48	0.73	3
Time point for established liaison with agencies and organisations affected on matters of strategic management (rescue service, police)	4.41	0.86	3
All time points for the updated references of patients to the receiving healthcare facilities	4.40	1.08	1
Time point and type of media contacts (including press releases, press communiqué, press conference)	4.32	0.77	2
Decisions on sending liaison officer to county administrative board	4.20	0.72	3
**Patient transport/resources**			No
Time point at which the first priority 1 (T1) patient was transported to healthcare facilities	4.68	0.76	1
Time point at which the last priority 1 (T1) patient was transported to receiving healthcare facilities	4.68	0.71	1
The number of transport units (other than by ambulance)	4.53	0.74	3
Alternative transport units	4.34	0.76	1
**Initial medical response management at the local level (healthcare facilities)**			No
Total number of patients received by health care facilities	4.96	0.19	2
Time when the alarm receiver on receiving hospital is alerted	4.85	0.36	1
Arrival time the first ambulance to the healthcare facility	4.79	0.49	1
Arrival time for the first priority 1 (T1) patient from the incident site to the healthcare facility	4.79	0.49	1
Arrival time for the last priority 1 (T1) patient from the incident site to the healthcare facility	4.72	0.52	1
Time point of startup / shutdown when decontamination station is in operation	4.70	0.60	1
Name of receiving hospital	4.68	0.60	1
Time point of established medical management coordination at receiving hospital (ie, when management can be exercised)	4.64	0.73	3
Change and time point for changing the emergency level at the healthcare facilities including startup / shutdown	4.59	0.93	2
Time point at which the hospital activates its disaster medical management plan	4.58	0.68	1
Time point when medical management at each healthcare facility is deactivated	4.53	0.74	3
Arrival time of the first self-presenting patient to the healthcare facility	4.41	0.77	1
**Injury severity and mortality (patient characteristics)**			No
The total number of injured	4.96	0.18	1
Mortality on scene (number)	4.90	0.30	1
Mortality in the emergency department (number)	4.89	0.31	2
Mortality Pre-hospital and during transport (number)	4.86	0.43	1
Dominating type of injury	4.72	0.52	1
Injury severity (ISS)	4.62	0.88	2
The total number of self-presenting patients to the healthcare facility	4.58	0.50	1
Affected but uninjured (estimated number)	4.37	0.97	2
**Staff equipment**			No
Disturbance and type of disturbance of communication	4.60	0.72	1
Disturbance and type of disturbance of documentation systems	4.50	0.82	1

* Acronym for the content, defined in the Major Incident and Medical Management Support Course (MIMMS) developed in Great Britain.

In Round 1 experts were asked to indicate their degree of agreement with each statement on a five-point Likert scale. The experts were also encouraged to include additional comments and/or statements that they considered were missing in the Round 1 questionnaire. The consensus level was set at 80%. No clear guidance for the level of consensus exists in the Delphi literature, but 75 % has been suggested to be a minimum [11]. However, due to the relatively small group of experts consensus was considered reached when 80% of the experts agreed on how important or non-important a statement was. For purposes of analysis, the five-point Likert scale was tricotomized to a three- point Likert scale with “1–2” representing totally disagree, “3” representing neutral and “4-5” representing totally agree, as suggested in other studies [18,20]. Data were analysed using SPSS statistics version 21 to measure central tendency (mean) and dispersion level (standard deviation, SD).

After analysing Round 1, minor modifications were made to some statements based on participant comments so as to improve clarity (n=20). Furthermore, statements that were considered not consistent with the aim of the study or were perceived as a replication (n=7) were withdrawn. After the Round 1 subsequent Rounds incorporated the results of feedback from the previous Round.

### Round 2

The questionnaire distributed in Round 2 comprised all statements that the experts had not reached consensus on (n=16) and the new statements suggested by the experts (n=37), in total 53 statements. Statements where experts had reached consensus in Round 1 (n= 44) were shown, but could no longer be graded. Round 2 incorporated feedback from Round 1 expressed as median scores from the panel as a whole, as well as each individuals own response from Round 1. Experts were asked to reconsider their initial opinions regarding uncertain statements from Round 1, and once again indicate their level of agreement regarding the new statements.

### Round 3

The 41 statements that the experts had not reached consensus on were distributed in the final Round 3. Feedback on the panel median and individual grading from Round 2 was provided for each expert.

## Results

Statements on which the experts reached or did not reach consensus are shown in Tables 3 and 4.

**Table 4:** Statements which did not reached expert consensus

**Area**	**Mean**	**SD**
**Initial medical response management at the regional (strategic) level**		
If DO assumes the role as initial medical management coordinator	4.32	0.94
Time point when regional medical command centre issues first situation overview	4.27	1.13
Time point when the last staff person notified reported to the appropriate location according to the disaster medical management plan (DMMP)	3.75	1.02
Name of duty officer	3.41	1.32
**Initial medical response management at the local level (scene of the incident)**		
Time point when first EMS ambulance arrive at the rendezvous point	4.25	0.96
The use of protective gear by the medical responders on scene	4.17	1.09
Ensure that the established actions for safety and health on scene are followed	4.07	1.18
The usage of triage tags	4.00	0.76
Time point at which the last patient has been triaged at scene of the MI	3.86	1.09
**Management/liaison (when nothing else is specified)**		
Decisions on common (regional) information efforts	4.24	0.78
Patient transport/resources		
Time point for the first secondary transport	4.07	0.76
Time point for the last secondary transport	4.03	0.83
**Initial medical response management at the local level (healthcare facilities)**		
Personal protective equipment used at the receiving healthcare facilities (for example chemical protection )	4.25	0.88
Time when first / last patient from scene of the incident was prioritised (after arrival at the receiving healthcare facility)	4.14	0.97
Name of recipient of information / alarms at the healthcare facilities	3.85	1.17
Triage System at receiving healthcare facilities	3.85	1.00
Number of patients who are over-or under-prioritised on arrival at the emergency department	3.50	0.96
Number of patients arriving per unit of time for the different receiving healthcare facilities (unit of time can be 5, 10 or 15 min)	3.10	1.22
When each patient leaves the emergency department	3.07	1.15
**Staff equipment**		
Used communication and documentation systems	4.10	0.81

### Round 1

**Figure 1:**
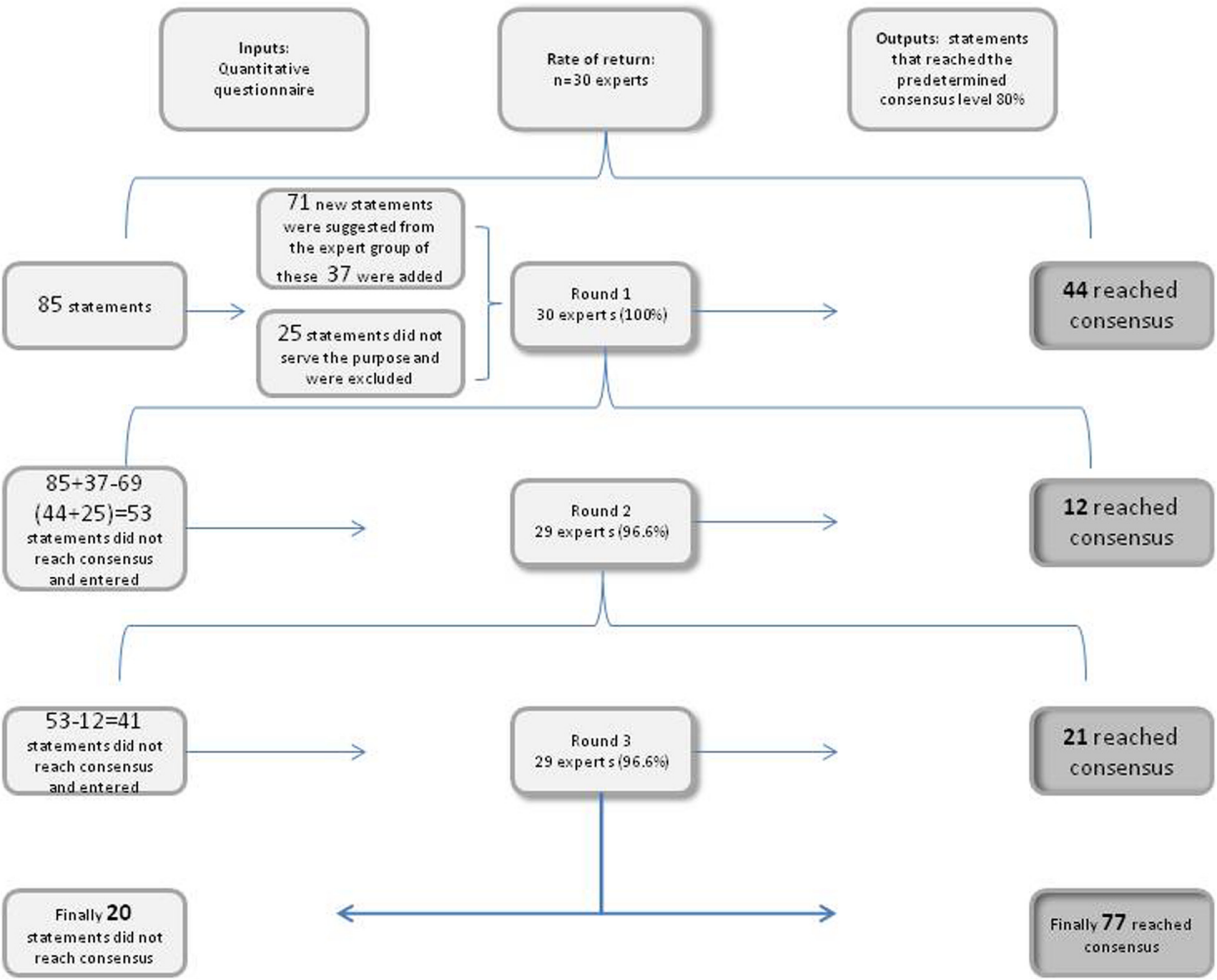
Overview of consensus process for indicators for disaster medical response.

After the first round, the experts generally agreed that data concerning the areas related to initial medical response management at the regional level and at the local level (at the scene of the incident and at the healthcare facilities) and type of incident, have an essential role in the activation of the DMR. Of the initially 85 predefined statements, 44 reached the predetermined consensus level of 80% (Figure 1). The experts stated notification, incident characteristics, first reports, coordination, alerting hospitals, mobilisation of transport, communications and information as being essential register data. However, there were some comments from the group concerning the number of points in time (too many), and that it is essential to also include content of reports, and these modifications were made prior to Round 2. These kinds of data are generally captured in the literature as important activities with impact on the initial DMR [15]. Furthermore, 18 statements, all related to type of incident, were condensed to a single statement. This resulted in a total of 25 statements that were withdrawn. The experts suggested 37 new statements considered consistent with the aim and these were included (Figure 1).

### Round 2

In the second, 37 new statements were formulated based on suggestions from the experts. Of these new statements 21% (8/37) reached expert consensus at Round 2. New statements mainly included additional time points, processes involved in triage and content of reports as well as of decisions. In the areas injury severity and mortality, as well as patients characteristics, all (8/8) statements reached expert consensus after Round 2 . Content of reports and decisions in the area initial medical response management at the local level (at the scene of the incident), and first unit on scene reached expert consensus. In total 12 statements reached expert consensus in Round 2. This Round rendered a response rate of 96.6% (n 29) and 12 additional statements reached the predetermined consensus level of 80%, leaving 41 statements that the experts had not reached consensus on (Figure 1).

### Round 3

After three Rounds, 77 statements had reached the predetermined consensus level and 20 had not (Figure 1). All 29 participants from Round 2 responded to Round 3, a response rate of 100%. In Round 3, 12 of 13 statements regarding the areas management/liaison, incident management in liaison with other agencies, and 26 of 31 statements regarding scene management reached expert consensus. Of the 37 statements suggested by the experts there were 29 included in round 3, and out of these 52% (15/29) reached consensus. In total after all three Rounds 79% (77/97) of the statements reached expert consensus with a mean rating varying between 4.20-4.96 (SD 0.18-1.10). Among the statements that did not reach expert consensus, initial medical management at health-care facilities received the lowest value 3.07 (SD 1.15). The mean for the 20 statements that did not reach expert consensus ranged from 3.07-4.32 (SD 0.76-1.32).

## Discussion

One strength of the Delphi method is that all experts have the same impact on the consensus process. The risk of influencing other participants is also reduced by avoiding face-to-face discussions [21]. The expert panel in the present study, although comprising participants with different fields of expertise, were treated as a homogenous group. In this respect, expert knowledge representing a variety of viewpoints can provide relevant inputs in the Delphi process, this can also help in minimising bias [11]. The majority of the participants have many years of experience in disaster medicine, and they are involved in these issues every day. From this perspective, their motivation to participate and their opinions and judgments can be seen as valid representation of the needs and requirements regarding standard data for reporting MI. The response rate was high, with 100 % in Round 1 and only one drop-out in Round 2. The high response rate could be due to the authors’ ongoing communication regarding the importance of each participant’s contribution, continual reminders and a limit set at three Rounds. It may also reflect the experts’ understanding of the importance of the study.

There seems to be a general consensus on the importance of data regarding the initial medical response, regarding processes, outcome as well as structure. This was no surprise since most experts in disaster medicine consider that it is during this initial phase that there is the greatest chance to influence the outcome of MIs [15]. It is reasonable to believe that adequate data from the first phase will provide clues on how to improve the DMR in the future. If important processes in this phase are documented and time logged this may well result in the finding of possible weak links in the early phase of the response chain, and also where communication and reporting need improvement. The fact that the expert panel commented the need for qualitative data such as content of reports, confirms the view that mere timelogs without procedure content have limited value. In accordance with the Delphi technique this was included in subsequent Rounds and consensus was also reached regarding several of these issues. It is interesting, however, that several experts commented on there being an abundance of timepoints, while others suggested the inclusion of even more. Furthermore, it was not until Round 2 that all data regarding patient characteristics reached expert consensus. Since it is a general belief that morbidity and mortality are the most importent outcomes when judging the effectiveness of the DMR, an explanation for these data not reaching consensus in the first Round could possibly lie in how the initial statement was formulated [8]. An interesting observation was the high number of statements suggested by the experts that reached general consensus 62% (23/37). One strength of the Delphi method is that it allows experts to influence the input of data, thereby adding important information that may previously have been overlooked. Round 3 resulted in just over half of the remaining statements reaching a consensus level of 51% (21/41). In this Round most statements regarding triage of patients reached expert consensus.

Triage is an important process in a DMR where, despite extensive research, more evidence-based facts are needed. Despite all efforts to include triage in textbooks, training programmes and exercises, there are few reports providing information on how triage is actually employed [22]. It is therefore surprising that consensus on inclusion of these data was only reached in Round 3. Perhaps future research based on qualitative methods may clarify issues on triage. There are similarities between internationally identified process indicators for collecting and reporting DMR. Most notably, less emphasis was placed on the use of triage system and protective equipment by the Swedish experts. The use of templates for collecting and reporting DMR can be used to determine where further teaching is required in medical response management, to enhance the planning and response to future events, and to see if processes contribute to the outcome. However, so far no studies on medical response management have reported results leading to a suggested template, which emphasizes the gap between DMR research and practice. Finally, the fact of the matter is that an international standard template for collecting and reporting DMR data is difficult since each country has its own DMR structure and an international data- reporting system may not be feasible. Even though, we have to start to identify accurate key indicators in the DMR performance. A majority of the study’s indicators are to a substantial extent identical to previous recommended by Debacker et al. [8]. This indicates that the DMR processes and general principles for response are based on the same fundamental activities despite differences in DMR structure and resources in different countries. The 77 statements will be presented to stakeholders on national level where the possibility for them to be included in training as well as in practice will be discussed and further developed.

### Significance of this study for the future

Based on the Delphi consensus technique this study has identified key indicators essential when registering events in the initial DMR, and provides guidance as to what to include in a national DMR register. Benchmarking, the collection of relevant data from many DMRs, allows comparison and the assessment of the strengths and weaknesses of the DMR management structure, leading to systematic and steady improvement.

### Limitations

A strategic selection of experts was made with a broad requirement for knowledge and experience in the field of disaster medicine. This selection was mainly based on the knowledge in the author-group dating back more than 20 years in disaster medicine on a national level, and was done considering who has the needed competences and was likely to answer the questionnaire. The word approximately is used in order to avoid an exact definition of what an expert is, which is very difficult to define. Considering the high response rate, we consider this to be a well-chosen strategy; since all invited did participate. According to Keeney, the exact composition of the expert group can affect the results obtained [11].

The first questionnaire was based on a review of the literature. This could have introduced bias by causing the participants to feel pressured to alter their view on DMR according to the authors’ predefined statements, even though they were given the opportunity to suggest new statements. Our attempts to describe statements in an unambiguous way may not have been successful in some aspects, as revealed by the experts’ comments. This emphasises the importance of ongoing discussion in order to reach clear and valid definitions, and the practical use of these terms as a base for management and performance in DMR [8,15]. True anonymity cannot be guaranteed as the experts were known to the authors and each other, which is described as “quasi-anonymity” [11]. Accepting this, the authors still believe that the experts’ awareness of each other’s participation, in the same research project, increased the response rate in this study. Although consensus was reached on 77 statements, we cannot be sure that the optimal key indicators have been identified only that they reflect expert consensus in a Delphi study. It is therefore important that one acknowledges the influence of bias and the validity of the results when using the Delphi technique [11].

There may also be additional results if a sub-group analysis was to be done. This may be the subject for further analysis, but according to the authors, will not improve present study where the aim was to find a national consensus on a broad basis.

## Conclusions

The Delphi technique can be used to achieve consensus on data, comprising key indicators that are essential for registering the response to major incidents and disasters.

– This study identified 77 key indicators essential for data reporting from the response of major incidents.

– Consensus at the national level can, in essential respects, be derived from the results of international studies.

– Future research will demonstrate whether the results from the present study can serve as a base for a generally acceptable national register, thus making participation and comparison in international studies more practical.

## Competing interest

The authors declare that no competing interest exists.

## Author’s contributions

Study design (MR, MJ, AR, MC ). Data collection and analysis (MR, MJ, AR). Manuscript writing (MR, MJ, AR). Contribution to the writing of the manuscript (MC, LS, DG). All authors have read and approved the final manuscript (MR, MJ, AR, MC, LS, DG).
